# The developmental trajectories of phubbing in Chinese adolescents: a variable-centered and person-centered perspective

**DOI:** 10.3389/fpsyt.2026.1784683

**Published:** 2026-06-10

**Authors:** Jun Zhao, Shoufeng Li

**Affiliations:** 1 Mental Health Education and Counseling Center, Nanchang Hangkong University, Nanchang, China; 2 School of Marxism , Nanchang Hangkong University, Nanchang, China; 3 School of Marxism , Nanjing University of Aeronautics and Astronautics, Nanjing, China

**Keywords:** adolescents, latent growth modeling, developmental trajectories, digital mental health, latent growth mixture modeling, mobile phone usage duration, phubbing

## Abstract

**Objectives:**

From both variable-centered and person-centered perspectives, the present study aimed to examine the developmental trajectories of phubbing among Chinese adolescents and to investigate whether mobile phone usage duration was associated with overall growth patterns and heterogeneous trajectory membership.

**Methods:**

Participants were 1,488 middle school students from Jiangxi Province, China. Data were collected across three waves at approximately four-month intervals. Phubbing was assessed using the Generic Scale of Phubbing, and mobile phone usage duration was measured at baseline in hours per week. Latent growth modeling was used to examine the average developmental trajectory of phubbing, and latent growth mixture modeling was conducted to identify heterogeneous trajectory subgroups. Multinomial logistic regression was further used to examine the association between mobile phone usage duration and trajectory subgroup membership.

**Result:**

The latent growth model showed that adolescent phubbing increased significantly over time, with substantial individual differences in both initial levels and rates of change. The latent growth mixture model identified four distinct trajectory groups: the Low-Level—Rising Group, the Medium-Level—Stable Group, the High-Level—Declining Group, and the Very High-Level—Sharp Decline Group. Mobile phone usage duration was positively associated with the initial level of phubbing but negatively associated with the rate of change. Longer mobile phone usage duration was also associated with a greater likelihood of membership in trajectory groups characterized by higher initial levels of phubbing.

**Conclusion:**

Findings revealed both an overall increase and meaningful heterogeneity in adolescent phubbing trajectories. Mobile phone usage duration appears to be an important but limited indicator of phubbing development, particularly for distinguishing adolescents with higher baseline phubbing. These findings provide a longitudinal basis for understanding adolescent phubbing and suggest the need for differentiated prevention strategies.

## 1 Introduction

In the digital age, mobile smart devices have become a salient ecological context shaping adolescents’ psychosocial development. During early and middle adolescence, individuals undergo rapid transitions in identity development, peer affiliation, emotional regulation, and social cognition. From a social investment perspective, developmental changes in social roles and interpersonal commitments may shape behavioral adjustment across adolescence (1). In light of the ongoing development of adolescents’ self-regulatory capacities and interpersonal adaptation skills, they may be particularly vulnerable to mobile phone-related behavioral problems and their psychosocial consequences (2). One increasingly prevalent behavior is phubbing, defined as ignoring interaction partners in face-to-face settings due to sustained attention to a mobile phone (3). Although smartphones provide adolescents with convenient access to communication, entertainment, learning resources, and peer interaction, excessive or poorly regulated mobile phone engagement may increase the likelihood that phone use intrudes into offline social interactions. Phubbing is not merely a matter of digital etiquette; rather, it represents a technology-mediated interpersonal behavior with important implications for adolescent social functioning and mental health. By diverting attention away from face-to-face communication, phubbing may reduce perceived responsiveness, impair communication quality, and weaken adolescents’ sense of being valued in interpersonal relationships. For adolescents, whose psychological adjustment depends heavily on peer acceptance, family support, and stable interpersonal feedback, frequent phubbing may contribute to interpersonal conflict, social avoidance, emotional maladjustment, and problematic smartphone-related behaviors (4–6). Prior studies have linked phubbing or related smartphone-distraction behaviors to poorer relationship quality, social exclusion, loneliness, psychological distress, anxiety, depressive symptoms, lower psychological well-being, and smartphone addiction (7–10). Recent studies have further strengthened the clinical and developmental relevance of this topic. For example, adolescent phubbing has been linked to depression through peer relationship quality and psychological need frustration (11), parental phubbing has been associated with adolescents’ depression and non-suicidal self-injury (12, 13), and longitudinal evidence suggests that parental phubbing may undermine adolescents’ core self-evaluation through basic psychological need satisfaction (14). Qualitative evidence from child and adolescent psychiatry also highlights phubbing as a clinically relevant phenomenon in digital mental health contexts (15). More broadly, systematic evidence suggests that digital communication technologies may shape the quality of family and interpersonal relationships, indicating that phone-related interpersonal disruption should be understood within broader relational contexts (16). Therefore, examining the developmental trajectories of adolescent phubbing is important not only for understanding how mobile phone-related interpersonal behaviors change over time but also for identifying adolescents who may follow different risk patterns and informing developmentally sensitive prevention efforts.

Mobile phone usage duration is a theoretically important but empirically contested antecedent of adolescent phubbing. On the one hand, longer phone use may increase adolescents’ exposure to phone-related cues, online social feedback, instant notifications, and habitual checking opportunities, thereby increasing the likelihood that mobile phone engagement intrudes into face-to-face interactions. Consistent with the habit formation perspective, repeated and prolonged phone engagement may gradually transform phone checking from a deliberate action into an automatic, context-triggered habit, providing a behavioral context in which phubbing is more likely to emerge and become reinforced (17, 18). This process is also consistent with broader reinforcement and habit-formation accounts, which suggest that behaviors maintained by intermittent rewards and repeated cue-response associations may become increasingly stable over time (19–21). In the smartphone context, empirical evidence also suggests that smartphone use can become habitual under relevant cues (22), and reinforcement-based perspectives have been widely used to understand repetitive engagement in potentially addictive behaviors (23). In addition, the I-PACE model suggests that technology-related behaviors may become persistent through interactions among cue reactivity, affective and cognitive responses, gratification, and reduced executive control (24, 25). From this perspective, longer phone use may increase the opportunities for phone-related cues and gratification processes to interfere with adolescents’ offline social interactions. Consistent with these theoretical assumptions, previous studies have suggested that frequent or excessive smartphone-related behaviors are associated with higher levels of phubbing and related problematic smartphone behaviors (3, 26, 27).

On the other hand, emerging evidence suggests that mobile phone usage duration may not necessarily lead to phubbing behavior, as the amount of phone use alone does not fully capture the interpersonal context or psychological meaning of smartphone engagement. Adolescents use mobile phones for diverse purposes, including learning, communication, entertainment, information seeking, and maintaining peer relationships (28, 29). Therefore, the same amount of phone use may have different psychosocial meanings depending on use motives, interaction contexts, self-regulation, and the degree of problematic or compulsive use. Some evidence suggests that phubbing is more strongly associated with smartphone addiction, fear of missing out, Internet addiction, self-control, social comparison, loneliness, social exclusion, and normative beliefs than with phone use time alone (3, 30–32). This view is consistent with the compensatory Internet use perspective, which emphasizes that problematic digital engagement should be understood in relation to users’ motives, needs, and psychosocial contexts rather than exposure time alone (33). In addition, evidence based on objectively recorded screen time indicates that the relationship between screen time and problematic smartphone or social network use may be relatively weak, suggesting that the quantity of phone use alone may not fully capture the psychological and interpersonal risks of smartphone engagement (34). Consistent with this view, a recent large-scale longitudinal cohort study found that high or increasing addictive screen use trajectories, rather than total screen time alone, were associated with suicidal behaviors, suicidal ideation, and poorer mental health among youths (35). These findings indicate that the association between mobile phone usage duration and phubbing may be more complex than a simple linear relationship and that developmental trajectory approaches are needed to clarify how mobile phone engagement is associated with adolescents’ psychosocial functioning.

Most existing research on phubbing has adopted cross-sectional designs, which limits conclusions about within-person change and developmental dynamics. Although a small number of longitudinal studies have begun to examine phubbing over time, many have relied primarily on repeated-measures ANOVA or cross-lagged approaches. These approaches are useful for testing mean differences or reciprocal associations, but they are less suitable for describing individual growth trajectories. Consequently, findings remain mixed regarding whether phubbing increases linearly, remains largely stable, or follows a curvilinear pattern during adolescence (36, 37). From a variable-centered perspective, latent growth modeling can estimate the average initial level and rate of change in phubbing while also capturing individual differences in these growth parameters (38). More importantly, this approach allows researchers to examine whether mobile phone usage duration predicts the intercept and slope of phubbing. In other words, it can clarify whether adolescents who spend more time using mobile phones show higher initial levels of phubbing and whether phone use duration is associated with subsequent changes in phubbing over time. However, variable-centered approaches are not sufficient for capturing the full developmental complexity of adolescent phubbing. Such approaches typically assume that individuals vary around a single average trajectory, which may conceal qualitatively distinct developmental patterns. In contrast, person-centered approaches emphasize identifying subgroups of individuals who share similar developmental trajectories, thereby capturing meaningful heterogeneity in development (39, 40). In developmental psychology, differentiated trajectories have been documented for a range of psychosocial characteristics, including internalizing and externalizing problems (41). Yet this perspective has been underutilized in phubbing research. It remains unclear whether adolescents cluster into distinct phubbing trajectory subgroups, such as low-rising, medium-stable, high-declining, or very high-sharply declining groups. Neglecting such heterogeneity risks ecological fallacy, whereby inferences about individual developmental processes are incorrectly drawn from aggregate group-level trends (42).

A person-centered perspective also provides a more nuanced way to examine the role of mobile phone usage duration. The effect of phone use duration on phubbing may not be limited to predicting the overall initial level or average rate of change. Rather, longer phone use may differentiate adolescents’ likelihood of belonging to distinct developmental subgroups. For example, adolescents with longer mobile phone usage duration may be more likely to follow trajectories characterized by different baseline levels or patterns of change because repeated phone engagement strengthens habitual attention to mobile devices. At the same time, some adolescents with initially high phubbing may later show declining trajectories, possibly because accumulated negative consequences, increased self-regulation, parental monitoring, or academic demands lead them to reduce phone-related interpersonal disruption. Therefore, examining mobile phone usage duration from both variable-centered and person-centered perspectives may provide a more comprehensive understanding of how phone use is associated with the development and heterogeneity of adolescent phubbing.

To address these gaps, the present study conducted a three-wave longitudinal survey over one academic year among Chinese middle school students. The study integrated variable-centered and person-centered approaches to examine the role of mobile phone usage duration in the developmental trajectories of adolescent phubbing. Specifically, latent growth modeling was used to estimate the average initial level and rate of change in phubbing and to examine whether mobile phone usage duration was associated with these growth parameters. Latent growth mixture modeling was then applied to identify heterogeneous phubbing trajectory subgroups, and the association between mobile phone usage duration and subgroup membership was further examined. Based on the above theoretical arguments and prior empirical findings, the present study proposed the following hypotheses:

Hypothesis 1. Adolescent phubbing would show significant developmental change over time, with substantial individual differences in both initial levels and rates of change.
Hypothesis 2. Longer mobile phone usage duration would be associated with higher initial levels of adolescent phubbing and with individual differences in the rate of change in phubbing over time.
Hypothesis 3. Adolescents would exhibit heterogeneous developmental trajectories of phubbing, characterized by distinct subgroups with different initial levels and patterns of change.
Hypothesis 4. Longer mobile phone usage duration would be associated with a greater likelihood of membership in distinct phubbing trajectory subgroups.

## 2 Materials and methods

### 2.1 Participants and procedures

The study employed a questionnaire-based longitudinal design with three waves of data collection. Participants were second-year middle school students from Jiangxi Province, China. A cluster sampling method was used, with intact classes as the sampling units. Specifically, students were recruited from 4 middle schools and 32 intact classes. Data collection began in September 2023, and follow-up assessments were administered at approximately four-month intervals during the academic year, yielding three measurement occasions: T1, T2, and T3.

Before data collection, approval was obtained from the Ethics Committee of the Mental Health Education and Counseling Center, Nanchang Hangkong University, and permission was obtained from the participating schools. Because all participants were minors, written informed consent was obtained from their parents or legal guardians, and assent was obtained from the adolescents themselves. Participation was voluntary, and students were informed that they could withdraw from the study at any time without penalty. The questionnaires were administered in classroom settings by trained research assistants using standardized instructions. During administration, students completed the questionnaires independently, and teachers were asked not to interfere with students’ responses. To protect confidentiality and match responses across waves, each participant was assigned a unique anonymous code. No personally identifying information was used in the data analysis.

The effective baseline sample comprised 1,488 adolescents at T1. At subsequent waves, 312 participants were absent at T2 and 340 participants were absent at T3. Eligibility criteria were as follows: (a) voluntary participation and willingness to complete the study procedures, and (b) being 12–16 years of age and currently enrolled in middle school. Exclusion criteria included having received psychological counseling or having been diagnosed with a mental disorder in the preceding six months. This information was obtained from participant reports during the screening process.

Attrition analyses indicated no evidence of selective dropout. Specifically, T1 phubbing scores did not differ significantly between adolescents who later attrited and those who remained in the study, lost: *M* = 2.04, *SD* = 0.89; retained: *M* = 1.98, *SD* = 0.82, *t* = 1.50, *p* = 0.135. These results suggest that attrition was unlikely to systematically bias the longitudinal estimates. At baseline, participants’ mean age was 13.56 years, *SD* = 0.76. The sample included 807 boys, 54.2%, and 681 girls, 45.8%.

### 2.2 Measurement

#### 2.2.1 Phubbing

Phubbing was measured using the Generic Scale of Phubbing, GSP, developed by Chotpitayasunondh and Douglas (5). The GSP consists of 15 items loading on four dimensions: interpersonal conflict, nomophobia, self-isolation, and problem acknowledgement. The GSP was selected because it is a widely used measure of general phone-snubbing behavior and has demonstrated good psychometric properties in previous studies. Responses were recorded on a 5-point Likert scale, with higher scores indicating more frequent phubbing.

The scale demonstrated excellent internal consistency across the three waves, Cronbach’s *α* = 0.94 at T1, 0.94 at T2, and 0.96 at T3. Confirmatory factor analyses supported the adequacy of the measurement model at each time point: T1, *χ*²/*df* = 4.51, RMSEA = 0.06, CFI = 0.94, TLI = 0.92; T2, *χ*²/*df* = 4.73, RMSEA = 0.06, CFI = 0.92, TLI = 0.90; and T3, *χ*²/*df* = 4.32, RMSEA = 0.05, CFI = 0.94, TLI = 0.92. Fit indices were interpreted using conventional cutoffs, with RMSEA values below 0.08 and CFI/TLI values close to or above 0.90 indicating acceptable model fit (43).

#### 2.2.2 Mobile phone usage duration

Mobile phone usage duration was assessed at baseline using a self-report item asking participants to report their average time spent using a mobile phone. Responses were recorded in hours per week, with higher scores indicating longer mobile phone usage duration. In the present study, mobile phone usage duration was treated as a time-invariant predictor of phubbing growth parameters and trajectory subgroup membership.

### 2.3 Data analysis

Data analyses were conducted in several steps. Descriptive statistics, internal consistency coefficients, attrition analyses, and bivariate correlations were first calculated using SPSS 26.0. Longitudinal measurement invariance, latent growth modeling, and latent growth mixture modeling were conducted using Mplus 8.3. Statistical significance was evaluated at *p* <.05.

Missing data were handled using full information maximum likelihood, FIML. FIML uses all available information to estimate model parameters under the assumption that data are missing at random. This approach is suitable for longitudinal data with incomplete repeated measurements and reduces bias compared with listwise deletion.

Model fit was evaluated using multiple indices, including *χ*²/*df*, RMSEA, CFI, TLI, and SRMR where applicable. In general, RMSEA values below 0.08 and CFI/TLI values close to or above 0.90 were considered indicative of acceptable model fit, whereas values closer to 0.95 indicated good fit (43).

#### 2.3.1 Longitudinal measurement invariance testing

Longitudinal measurement invariance of the Generic Scale of Phubbing was examined across the three waves before conducting growth analyses. Longitudinal invariance evaluates whether the construct is measured equivalently across repeated assessments, thereby supporting meaningful comparisons of latent means and change over time. Following established recommendations, invariance was tested in a hierarchical sequence, including configural invariance, metric invariance, scalar invariance, and residual invariance models (44). Changes in model fit indices, particularly ΔCFI and ΔRMSEA, were used to evaluate whether additional equality constraints resulted in meaningful deterioration of model fit. Following commonly used criteria, changes of ΔCFI ≤.010 and ΔRMSEA ≤.015 were considered evidence that the more constrained model did not substantially worsen model fit.

#### 2.3.2 Latent growth modeling

Latent growth modeling, LGM, was employed to characterize the developmental trajectory of phubbing across the three waves (45). LGM is a structural equation modeling approach for longitudinal data that represents repeated observations as indicators of underlying growth factors. Specifically, the model estimates an intercept factor, reflecting individuals’ initial level of the construct, and a slope factor, capturing systematic change over time.

In the unconditional LGM, the factor loadings of the intercept were fixed to 1 across all three waves, and the factor loadings of the linear slope were fixed to 0, 1, and 2 for T1, T2, and T3, respectively. The means of the intercept and slope factors were estimated to describe the average initial level and average rate of change in phubbing. The variances of the intercept and slope factors were estimated to determine whether there were significant individual differences in baseline levels and rates of change.

After estimating the unconditional LGM, a conditional LGM was specified to examine whether mobile phone usage duration predicted phubbing growth parameters. In this model, mobile phone usage duration was entered as a time-invariant predictor of both the intercept and slope factors. This analysis allowed us to test whether adolescents with longer mobile phone usage duration showed higher initial levels of phubbing and whether phone usage duration was associated with individual differences in the rate of change in phubbing over time. Age and gender were included as covariates in the conditional growth model.

#### 2.3.3 Latent growth mixture modeling

Latent growth mixture modeling, LGMM, was used to examine heterogeneity in the longitudinal trajectories of adolescent phubbing from a person-centered perspective. LGMM extends conventional LGM by allowing the population to comprise a finite number of latent classes, each characterized by distinct growth parameters, such as class-specific intercepts and slopes. In doing so, LGMM identifies subgroups of individuals who follow qualitatively different developmental patterns over time.

In the present study, a series of unconditional LGMMs were estimated to identify the optimal number of latent trajectory classes for phubbing. Models with increasing numbers of classes were compared sequentially. Model selection was based on statistical fit, classification quality, parsimony, class size, model convergence, and substantive interpretability. Specifically, lower values of the Akaike information criterion, AIC, Bayesian information criterion, BIC, and sample-size-adjusted Bayesian information criterion, aBIC, indicated better relative fit. Entropy was used to evaluate classification accuracy, with higher values indicating clearer classification. The Lo–Mendell–Rubin likelihood ratio test, LMR, and the bootstrap likelihood ratio test, BLRT, were used to compare whether a model with *k* classes provided a significantly better fit than a model with *k* − 1 classes.

Given the reviewer’s concern regarding the stability of small classes, additional attention was paid to class size and theoretical interpretability. Models were not selected solely on the basis of information criteria. Instead, the final class solution was retained only when it showed acceptable classification quality, meaningful and interpretable trajectory patterns, adequate convergence, and no clearly superior alternative solution. To reduce the risk of local maxima, models were estimated using multiple sets of random starting values.

After the optimal class solution was identified, mobile phone usage duration was examined as a predictor of trajectory subgroup membership. Specifically, multinomial logistic regression was conducted to test whether mobile phone usage duration was associated with the likelihood of belonging to different phubbing trajectory subgroups. The Low-Level—Rising Group was used as the reference class. Odds ratios, ORs, and 95% confidence intervals were reported to indicate the direction and magnitude of the association between mobile phone usage duration and subgroup membership.

## 3 Results

### 3.1 Common method bias test

Common method variance (CMV) was assessed at each time point using Harman’s single-factor test. Exploratory factor analysis indicated that the extracted factors met the criterion of eigenvalues greater than 1, and the first unrotated factor accounted for 19.98%, 22.07%, and 20.56% of the variance at T1, T2, and T3, respectively. All values were below the conventional 40% threshold. These results suggest that CMV was unlikely to pose a serious threat to the interpretation of the study findings (46).

### 3.2 Descriptive statistics and correlations

**Table 1** presents the descriptive statistics and bivariate correlations among the study variables. Age was modestly and positively associated with mobile phone usage duration, *r* = 0.13, *p* <.001, but it was not significantly related to phubbing at any assessment wave. Mobile phone usage duration was positively correlated with phubbing across all three waves, with the strongest association observed for T1 phubbing, *r* = 0.27, *p* <.001. Phubbing also demonstrated significant temporal continuity, with correlations across waves ranging from *r* = 0.41 to 0.52, all *p*s <.001.

**Table 1:** Means, standard deviations, and correlation results of the study variables (*N* = 1488).

Variables	1	2	3	4	5
1 Age	1				
2 Mobile Phone Usage Duration	0.13^***^	1			
3 T1 Phubbing	0.06	0.27^***^	1		
4 T2 Phubbing	0.06	0.17^***^	0.52^***^	1	
5 T3 Phubbing	0.04	0.13^***^	0.41^***^	0.45^***^	1
Mean(*M*)	13.56	6.74	2.00	2.04	2.09
Standard Deviation(*SD*)	0.76	1.39	0.85	0.90	0.95

*p < 0.05, **p < 0.01, ***p < 0.001; Mobile phone usage duration unit: hours/week.

### 3.3 Longitudinal measurement invariance of phubbing

As shown in **Table 2**, the measurement model for phubbing demonstrated acceptable fit, *χ*² = 3106.230, *df* = 879, RMSEA = 0.041, CFI = 0.913, TLI = 0.902. Tests of longitudinal measurement invariance further indicated little to no deterioration in model fit across increasingly constrained models. Specifically, changes in fit indices between nested models were small, with ΔRMSEA = 0.000 and ΔCFI ranging from −0.001 to −0.004. These results supported the longitudinal measurement invariance of the phubbing scale across the three waves.

**Table 2:** Measurement invariance results of phubbing (*N* = 1488).

Variable	Model	*χ^2^*	*df*	RMSEA	CFI	TLI	ΔRMSEA	ΔCFI
Phubbing	M1	3106.230	879	0.041	0.913	0.902		
	M2	3152.395	901	0.041	0.912	0.903	0.000	-0.001
	M3	3289.037	931	0.041	0.908	0.902	0.000	-0.004
	M4	3354.139	959	0.041	0.907	0.904	0.000	-0.001

M1 is the configural invariance model; M2 is the weak invariance model; M3 is the strong invariance model; M4 is the strict invariance model.

### 3.4 Latent growth model of adolescent phubbing

The results of the linear latent growth model (LGM) for phubbing are reported in **Table 3**. Regarding model fit, the model demonstrated near-perfect fit, with *χ*²/df = 0.12, RMSEA = 0.000, and CFI and TLI approaching 1.00. Given that the model included only three repeated measures, this pattern may reflect a saturated or near-saturated model, in which global fit indices provide limited diagnostic information. Nevertheless, the estimated growth parameters remain interpretable for describing the mean initial level and rate of change in phubbing.

**Table 3:** Fit information and parameters of the latent growth model (*N* = 1488).

Variable	*χ* ^2^ */df*	RMSEA	CFI	TLI	Mean		Variance	
					Intercept	Slope	Intercept	Slope
Phubbing	0.12	0.00	1.00	1.00	30.10^***^	0.68^***^	11.57^***^	17.20^***^

*p < 0.05, **p < 0.01, ***p < 0.001.

**Figure 1:**
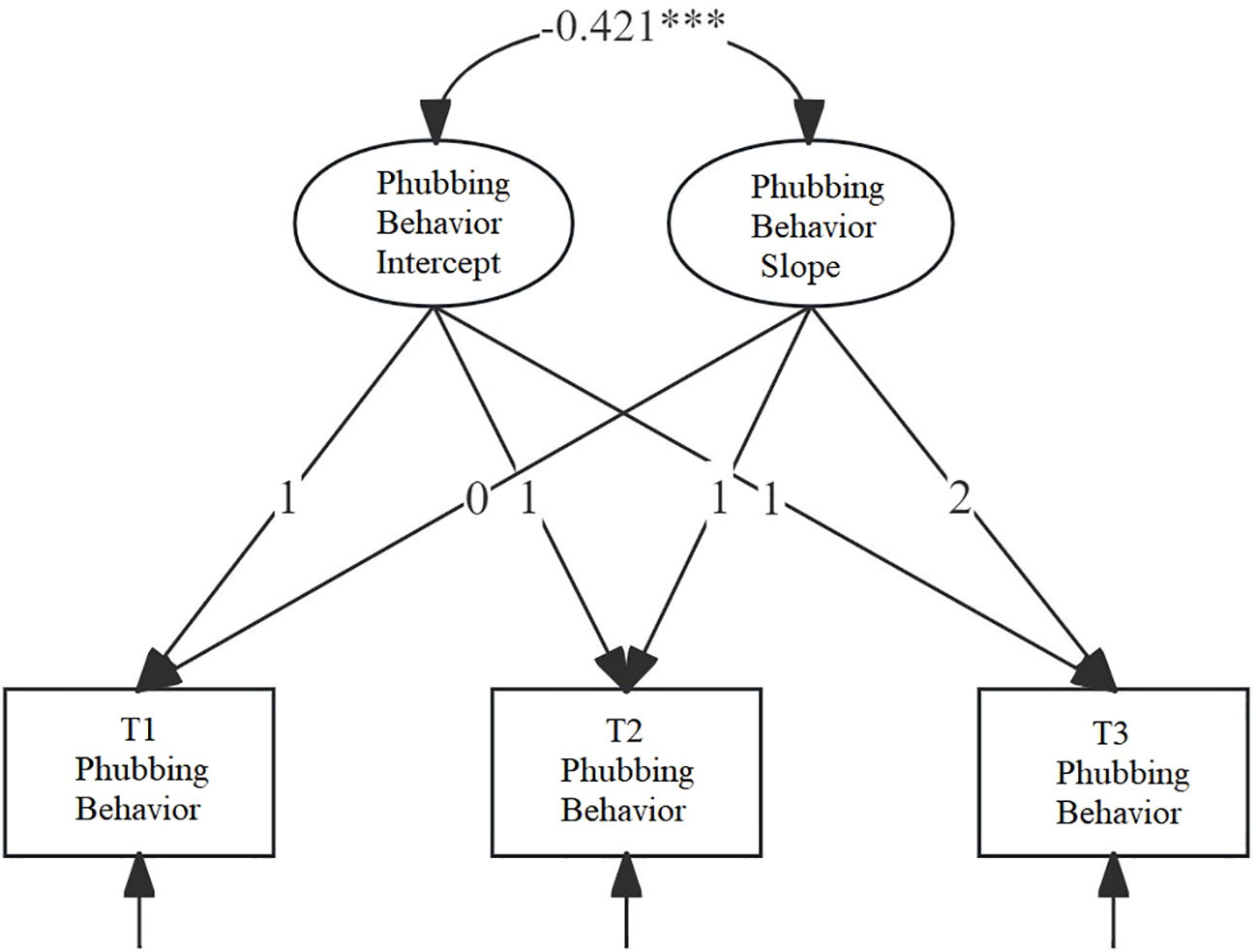
Unconditional latent growth model of adolescent phubbing.

Regarding growth parameters, the estimated mean intercept for phubbing was 30.10, *p* <.001, and the mean slope was 0.68, *p* <.001, indicating a significant increase in phubbing over time. The intercept variance was 11.57, *p* <.001, and the slope variance was 17.20, *p* <.001, suggesting substantial between-person variability in both baseline levels and rates of change. In addition, the intercept and slope were negatively correlated, *r* = −0.42, *p* <.001, indicating that adolescents with higher initial phubbing tended to show a slower subsequent increase. Taken together, these findings supported Hypothesis 1, indicating that adolescent phubbing showed significant developmental change over time and substantial individual differences in both initial levels and rates of change. The corresponding model is illustrated in **Figure 1**.

### 3.5 Latent growth mixture model of adolescent phubbing

The significant variances of the intercept and slope in the unconditional LGM indicated substantial individual differences in both the initial level and rate of change in adolescent phubbing. To further examine whether these individual differences reflected qualitatively distinct developmental patterns, latent growth mixture modeling (LGMM) was used to identify heterogeneous trajectory subgroups of phubbing.

Latent growth mixture models with two to five classes were estimated sequentially, and the model fit information is presented in **Table 4**. The four-class solution was retained as the final model based on a combination of statistical fit, classification quality, parsimony, class size, and substantive interpretability. Although the five-class model showed slightly lower AIC, BIC, and aBIC values than the four-class model, its entropy was lower, 0.73 versus 0.83, and both the LMR and BLRT tests were not significant. This indicates that the five-class solution did not provide a statistically superior improvement over the four-class solution. In contrast, the four-class model showed acceptable classification quality, significant LMR and BLRT tests, and clearly interpretable trajectory patterns. Therefore, the four-class solution was retained.

**Table 4:** Fit information summary of the latent growth mixture model (LGMM) (*N* = 1488).

Variables	Number of classes	AIC	BIC	aBIC	Entropy	LMR(*p*)	BLRT(*p*)	Group proportions
Phubbing	2	29590.85	29649.21	29614.26	0.64	0.012	<0.001	75.3%/24.7%
	3	29461.91	29536.18	29491.71	0.76	0.009	<0.001	2.6%/54.0%/43.4%
	4	29367.54	29457.73	29403.73	0.83	0.036	<0.001	35.6%/2.7%/41.7%/20.0%
	5	29348.33	29454.44	29390.90	0.73	0.824	1.000	16.5%/3.2%/2.0%/23.5%/54.8%

It should be noted that one class in the four-class solution accounted for only 2.7% of the sample. This class was retained because it represented a distinct trajectory pattern characterized by a very high initial level of phubbing followed by a sharp decline. However, given its small size, this class should be interpreted cautiously and should be further examined in future studies with additional waves and independent samples.

**Figure 2:**
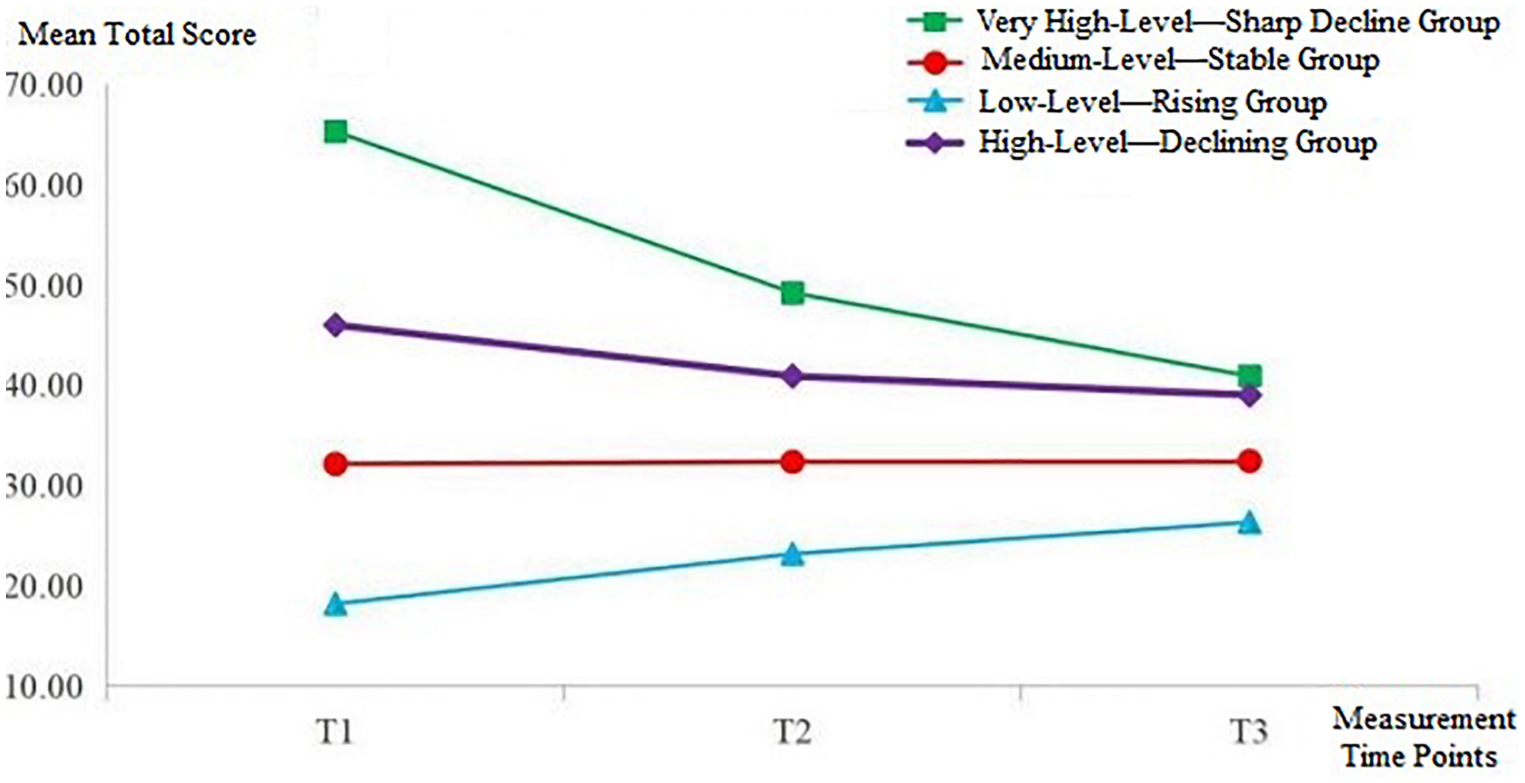
Developmental trajectories of phubbing for each heterogeneous subgroup across three measurement time points.

Parameter estimates for the four identified phubbing trajectories are presented in **Table 5**. Class 1 had a moderate initial level and a non-significant slope, intercept = 31.98, slope = 0.17, *p* = .559, and was labeled the “Medium-Level—Stable Group.” Class 2 had a very high initial level and a significant negative slope, intercept = 64.64, slope = −12.44, *p* <.001, and was labeled the “Very High-Level—Sharp Decline Group.” Class 3 had a low initial level and a significant positive slope, intercept = 18.50, slope = 4.16, *p* <.001, and was labeled the “Low-Level—Rising Group.” Class 4 had a high initial level and a significant negative slope, intercept = 45.29, slope = −3.59, *p* <.001, and was labeled the “High-Level—Declining Group.” The proportions of participants classified into the four groups were 35.6%, 2.7%, 41.7%, and 20.0%, respectively. The corresponding trajectory patterns are depicted in **Figure 2**. Therefore, Hypothesis 3 was supported, as adolescents exhibited heterogeneous developmental trajectories of phubbing with distinct initial levels and patterns of change.

**Table 5:** Parameters of different developmental trajectories of phubbing (*N* = 1488).

Variable	Trajectory class	Mean of intercept	SE	*p*	Mean of slope	SE	*P*
Phubbing	Class 1	31.98	0.46	<0.001	0.17	0.33	0.559
	Class 2	64.64	2.61	<0.001	−12.44	2.07	<0.001
	Class 3	18.50	0.24	<0.001	4.16	0.31	<0.001
	Class 4	45.29	0.74	<0.001	−3.59	0.58	<0.001

### 3.6 Mobile phone usage duration as a predictor of phubbing trajectories

A conditional LGM was specified to examine whether mobile phone usage duration was associated with the overall developmental trajectory of phubbing after controlling for age and gender. The model showed acceptable fit, *χ*²/*df* = 0.74, RMSEA = 0.000, CFI = 1.00, TLI = 1.00. With respect to growth parameters, mobile phone usage duration was positively associated with the intercept of phubbing, *β* = 0.32, *p* <.01, indicating that longer weekly mobile phone use was associated with higher initial levels of phubbing. In contrast, mobile phone usage duration was negatively associated with the slope, *β* = −0.21, *p* <.01, suggesting that longer weekly mobile phone use was associated with a slower rate of increase in phubbing across waves. These findings supported Hypothesis 2, showing that mobile phone usage duration was associated with both the initial level and rate of change in phubbing, although the association with the slope was negative.

To further examine whether mobile phone usage duration differentiated latent trajectory membership, a multinomial logistic regression was conducted with the “Low-Level—Rising Group” as the reference class and the other developmental trajectories as comparison groups. Greater mobile phone usage duration was associated with higher odds of being classified into the “Medium-Level—Stable Group,” OR = 1.06, 95% CI [1.04, 1.08], the “High-Level—Declining Group,” OR = 1.07, 95% CI [1.05, 1.09], and the “Very High-Level—Sharp Decline Group,” OR = 1.09, 95% CI [1.07, 1.12]. These results indicate that longer weekly mobile phone use was associated with a greater likelihood of membership in trajectory groups characterized by higher baseline phubbing relative to the Low-Level—Rising Group. These findings supported Hypothesis 4, indicating that mobile phone usage duration was associated with trajectory subgroup membership, particularly with a greater likelihood of belonging to groups characterized by higher baseline phubbing.

## 4 Discussion

This study used a three-wave longitudinal design to examine the developmental trajectories of phubbing among Chinese adolescents and to investigate the role of mobile phone usage duration from both variable-centered and person-centered perspectives. Four main findings emerged. First, adolescent phubbing showed a significant upward trend across the three measurement occasions, with significant individual differences in both initial levels and rates of change. Second, the LGMM results identified four heterogeneous developmental trajectories: Low-Level—Rising Group, Medium-Level—Stable Group, High-Level—Declining Group, and Very High-Level—Sharp Decline Group. Third, mobile phone usage duration was positively associated with the initial level of phubbing but negatively associated with the rate of change. Fourth, longer mobile phone usage duration was associated with higher odds of membership in trajectory groups characterized by higher initial levels of phubbing.

### 4.1 Average developmental trend of adolescent phubbing

The unconditional LGM showed that adolescent phubbing increased significantly across the three waves. This finding indicates that phubbing may become more frequent during the middle school period. From a developmental perspective, this pattern is understandable. Early and middle adolescence are periods in which peer interaction, autonomy seeking, and digital communication become increasingly salient. Mobile phones are not only entertainment tools but also important media for peer communication, social coordination, information seeking, and identity expression. Therefore, as adolescents’ mobile phone engagement becomes more embedded in daily life, the likelihood that phone use intrudes into face-to-face interactions may also increase.

This finding is consistent with the ecological view that adolescent behavior develops through continuous transactions between individuals and their social contexts. Although this study did not directly measure peer norms or family environments, developmental systems theory suggests that behavior should be understood as emerging from repeated interactions between adolescents and their everyday contexts (47, 48). In the present study, the observed increase in phubbing may reflect the growing integration of mobile phones into adolescents’ social ecology. However, this interpretation should be understood as a developmental explanation rather than direct evidence of specific contextual mechanisms.

The upward trend may also be understood from a reinforcement perspective. Phone checking in social situations may be maintained by intermittent social rewards, such as messages, likes, comments, or notifications, which can strengthen repeated engagement over time (19, 20, 23). Recent evidence also suggests that smartphone use may show habitual characteristics under relevant cues (22), and adolescent research indicates that exposure to phubbing may be associated with adolescents’ own phubbing behavior through social exclusion and self-regulation processes (31). The significant variances of both the intercept and slope are equally important. These findings indicate that adolescents differed not only in their initial levels of phubbing but also in how their phubbing changed over time. Thus, although the average trajectory increased, this average pattern does not necessarily describe every adolescent. This result provides the empirical basis for using a person-centered approach to examine whether distinct developmental subgroups exist.

From an effect-size perspective, the mean slope was statistically significant, indicating a reliable upward trend. However, the practical meaning of this increase should be interpreted cautiously. The finding does not imply that all adolescents experienced a severe increase in phubbing. Rather, it suggests a modest but systematic group-level increase across the school year. The significant slope variance suggests that the more practically meaningful issue may be the heterogeneity of developmental change, given that different adolescents followed different patterns around the average trend.

### 4.2 Heterogeneous developmental trajectories of phubbing

The LGMM identified four distinct developmental trajectories of adolescent phubbing. This finding supports the value of a person-centered approach. Whereas the unconditional LGM described the average developmental trend, the LGMM revealed that this average trend was composed of multiple subgroup-specific patterns. This is consistent with person-oriented developmental research, which argues that group-level averages may conceal qualitatively different developmental pathways within a population (39, 40, 42).

The largest subgroup was the Low-Level—Rising Group. Adolescents in this group started with relatively low levels of phubbing but showed a clear increase over time. This pattern suggests that low initial phubbing does not necessarily imply long-term low risk. From a developmental standpoint, this group is particularly important because it indicates that phubbing may emerge or intensify during the middle school period for a substantial proportion of adolescents. In other words, phubbing should not be understood only as a stable problem among adolescents who already show high levels of phone-related interpersonal distraction; it may also represent a developing behavioral pattern among adolescents who initially appear to be at relatively low risk. The identification of this group therefore highlights the value of longitudinal monitoring, as cross-sectional assessments may overlook adolescents whose phubbing is still at a low level but is increasing over time. From a prevention perspective, this group may be especially relevant because early guidance on phone-use boundaries, interpersonal awareness, and digital etiquette could be implemented before phubbing becomes more habitual or disruptive.

The Medium-Level—Stable Group maintained a moderate and relatively stable level of phubbing. This pattern suggests that, for a substantial proportion of adolescents, phubbing remained relatively consistent across the school year. Rather than indicating rapid escalation or reduction, this trajectory reflects a stable behavioral pattern at a moderate level. The value of identifying this group lies in showing that adolescent phubbing is not limited to high-risk or rapidly changing trajectories; for many adolescents, phone-related interpersonal distraction may already be embedded in everyday social interactions in a relatively persistent form. Although the level of phubbing in this group was not as high as that in the high-initial groups, its stability should not be overlooked. A moderate but enduring pattern of phubbing may still affect the quality of face-to-face communication, interpersonal responsiveness, and peer or family interaction over time. From a prevention perspective, this group may benefit less from intensive corrective intervention and more from maintenance-oriented guidance, such as strengthening awareness of phone-use boundaries, improving interpersonal attentiveness, and preventing moderate phubbing from becoming normalized or further intensified.

The High-Level—Declining Group began with a relatively high level of phubbing but showed a gradual decrease. This pattern indicates that elevated phubbing is not necessarily fixed. Some adolescents with high initial phubbing may reduce their behavior over time. The identification of this group is valuable because it shows that high initial phubbing does not inevitably develop into a persistent or worsening pattern. Instead, some adolescents may show behavioral adjustment across the school year, suggesting that phubbing trajectories can be reversible or developmentally flexible. From a practical perspective, adolescents in this group may benefit from support that consolidates positive behavioral change, such as strengthening awareness of interpersonal consequences and maintaining clearer boundaries between mobile phone use and face-to-face interaction. This finding should be interpreted carefully, however. The observed decline may reflect regression toward the mean, changes in mobile phone use patterns, increased awareness of interpersonal consequences, or other unmeasured contextual influences.

The Very High-Level—Sharp Decline Group represented a small subgroup with an initially very high level of phubbing followed by a marked decrease. Because this group accounted for only 2.7% of the sample, its interpretation requires caution. Although the class was retained because it showed a distinct trajectory pattern and the four-class solution was statistically and substantively interpretable, the small class size limits the certainty and generalizability of this subgroup. Nevertheless, the identification of this group is still informative because it suggests that a small proportion of adolescents may experience extreme but rapidly changing patterns of phubbing. Such a trajectory would be difficult to detect using only average-level analyses, which may obscure small subgroups with atypical developmental patterns. From a developmental perspective, this group indicates that even very high initial phubbing may not necessarily remain stable, and that sharp behavioral change can occur within a relatively short period. From an applied perspective, this subgroup may be particularly important for early identification, because adolescents with extremely high initial phubbing may require closer monitoring even if their behavior later declines. However, given the small class size and the limited number of assessment waves, this trajectory should be viewed as preliminary evidence of possible extreme developmental heterogeneity rather than a stable typology.

Overall, the LGMM findings show that adolescent phubbing is developmentally heterogeneous. The practical implication is that interventions should not assume a single uniform pathway. Adolescents with low but rising phubbing may require early preventive guidance, whereas adolescents with moderate-stable or high-initial patterns may require strategies focused on awareness, interpersonal consequences, and self-regulation.

### 4.3 Mobile phone usage duration and the average developmental trajectory of phubbing

The conditional LGM showed that mobile phone usage duration was positively associated with the intercept of phubbing. This means that adolescents who reported longer weekly mobile phone use tended to show higher initial levels of phubbing. Longer mobile phone use may increase exposure to phone-related cues, notifications, online feedback, and opportunities for phone checking. The I-PACE model suggests that technology-related behaviors may become persistent through interactions among cue reactivity, affective and cognitive responses, gratification processes, and executive control (24, 25). Applied to phubbing, adolescents who spend more time using mobile phones may encounter more phone-related cues and gratification processes, making it more likely that phone engagement occurs during offline interaction.

At the same time, this finding should be interpreted in light of evidence showing that problematic smartphone engagement is associated with both behavioral exposure and psychosocial vulnerability (4). The compensatory Internet use perspective also cautions against treating usage duration as equivalent to problematic use, because the meaning of digital engagement depends on users’ motives, needs, and psychosocial contexts (33). Therefore, longer mobile phone usage duration may indicate greater exposure to phone-related cues, but it should not be interpreted as sufficient evidence of problematic use or phubbing risk by itself. Recent evidence further suggests that the risk associated with digital engagement may depend not only on the amount of use but also on whether use becomes addictive or dysregulated. Neurocognitive evidence indicates that internet/smartphone addiction may involve altered cognitive control and reward-related processes (49), and evidence also suggests that habits can make smartphone use pervasive in daily contexts (50). In addition, a large longitudinal cohort study found that high and increasing addictive screen use trajectories were associated with suicidal behaviors, suicidal ideation, and worse mental health among youths (35).

The effect size should be interpreted in a balanced way. The standardized coefficient suggested that mobile phone usage duration was moderately associated with the initial level of phubbing. This represents a moderate association, suggesting that mobile phone usage duration is a meaningful predictor of adolescents’ initial phubbing levels. At the same time, it is not large enough to suggest that phone use duration alone explains phubbing. Phubbing is likely shaped by multiple factors, including self-control, smartphone use motives, fear of missing out, peer norms, family rules, and the perceived acceptability of phone use during social interaction. Thus, phone use duration should be regarded as an important but partial indicator of phubbing risk.

Mobile phone usage duration was negatively associated with the slope of phubbing. This finding indicates that adolescents with longer weekly phone use showed a slower rate of increase in phubbing over time. This result may appear counterintuitive if one assumes that longer phone use should always lead to faster increases in phubbing. However, it is consistent with the negative correlation between the intercept and slope in the unconditional LGM: adolescents with higher initial phubbing tended to show less subsequent increase. One plausible explanation is a ceiling or saturation pattern, whereby adolescents with longer phone use already had relatively high phubbing levels at baseline, leaving less room for further increase across three waves. Compared with its association with the initial level of phubbing, the association between mobile phone usage duration and the rate of change was relatively weaker. This suggests that mobile phone usage duration was more strongly associated with where adolescents started than with how quickly they changed. In practical terms, weekly phone use may be more useful for identifying adolescents who already show higher phubbing levels than for predicting rapid escalation. This distinction is important because it prevents overinterpreting phone use duration as a simple driver of worsening phubbing. Instead, the results suggest a more nuanced developmental pattern: longer phone use is associated with higher initial phubbing, but not necessarily with faster growth.

### 4.4 Mobile phone usage duration and trajectory subgroup membership

The multinomial logistic regression results showed that mobile phone usage duration was associated with membership in different phubbing trajectory subgroups. Compared with the Low-Level—Rising Group, longer weekly mobile phone use was associated with higher odds of belonging to the Medium-Level—Stable Group, the High-Level—Declining Group, and the Very High-Level—Sharp Decline Group. These results suggest that adolescents with longer mobile phone use were more likely to belong to trajectory groups characterized by higher initial levels of phubbing.

This finding is consistent with the conditional LGM result showing that phone use duration was positively associated with the intercept of phubbing. Both analyses suggest that mobile phone usage duration is more closely related to baseline differences in phubbing than to simple linear increases over time. From the perspective of the I-PACE model, longer phone use may reflect greater exposure to phone-related cues and gratification-based reinforcement, which may increase the likelihood that phone checking becomes integrated into offline social contexts (24, 25). From the habit formation perspective, repeated phone engagement may also contribute to more stable phone-checking routines, which may help explain why longer phone use was associated with membership in higher-initial trajectory groups (17, 18).

The odds ratios ranged from 1.06 to 1.09. These effects were statistically significant but modest in magnitude. This means that each additional unit of weekly mobile phone usage duration was associated with a small increase in the odds of belonging to the higher-initial phubbing trajectory groups. Although these effects are not large, they may still have practical relevance because mobile phone use is highly prevalent among adolescents. Small increases in odds may be meaningful when considered at the population level, especially for school-based screening and prevention. However, the modest size of the odds ratios also indicates that phone use duration should not be used as the sole basis for identifying risk.

The trajectory membership findings further show that the same predictor can be associated with different developmental pathways. Longer mobile phone use was not linked only to one high-risk pattern; rather, it was associated with multiple groups that differed in stability or decline but shared relatively higher initial phubbing levels. This suggests that phone use duration may help distinguish adolescents with different starting points, but it cannot fully explain why their subsequent trajectories diverge. This is a key limitation of relying on a single predictor. Future studies should incorporate broader individual and contextual variables to explain why adolescents with similar levels of phone use may follow different phubbing trajectories. In particular, self-regulation capacity (51), self-efficacy-related processes (52), and motivational needs for autonomy, competence, and relatedness (53, 54) may help explain why some adolescents maintain, increase, or reduce phubbing over time.

### 4.5 Practical implications

The findings have several implications for prevention and education. To begin with, the average increase in phubbing suggests that middle school may be an important period for guiding adolescents’ mobile phone use in interpersonal contexts. Preventive efforts should not focus only on reducing screen time but should also address when, where, and how mobile phones are used during face-to-face interaction. For example, school-based digital literacy programs may help adolescents recognize how phone checking affects communication quality and interpersonal respect.

Moreover, the identification of heterogeneous trajectories suggests that prevention should be differentiated. Adolescents in the Low-Level—Rising Group may benefit from early preventive guidance before phubbing becomes more frequent. Adolescents in higher-initial groups may require more focused support in developing phone-use boundaries during interpersonal communication.

In addition, mobile phone usage duration may serve as a simple and observable screening indicator. Because weekly phone use was associated with higher initial phubbing and greater odds of membership in higher-initial trajectory groups, it may help educators identify adolescents who could benefit from further assessment. Nevertheless, the modest effect sizes indicate that phone use duration alone is insufficient. It should be combined with other indicators, such as problematic smartphone use, self-control, peer norms, family communication, and psychological adjustment.

## 5 Limitations and future directions

Several limitations should be noted. First, the present study focused on mobile phone usage duration as the only primary variable of interest. Although it was significantly associated with phubbing growth parameters and trajectory membership, the modest effect sizes suggest that adolescent phubbing development is likely shaped by additional individual and contextual factors, such as smartphone use motives, compensatory use motives (33), fear of missing out, self-regulation (51), self-efficacy-related processes (52), peer norms, parental monitoring, motivational needs (54), and school context. Second, this study did not include mental health outcome variables. Although recent studies have linked phubbing or related smartphone-distraction behaviors to depression, non-suicidal self-injury, and broader mental health risks among adolescents (12, 13, 35), the present study could not determine whether different phubbing trajectories were associated with later mental health outcomes. Third, family, peer, and school contextual variables were not measured; therefore, the mechanisms underlying trajectory differences remain unclear. Fourth, the three-wave design allowed us to estimate linear change but limited the examination of nonlinear trajectories and the longer-term stability of developmental patterns. Finally, all measures were based on self-report, which may introduce reporting bias. Future research should replicate the identified trajectory patterns in larger and longer-term samples and combine self-reports with parent, peer, teacher, or objective smartphone-use data.

## 6 Conclusion

This study contributes to the literature by examining adolescent phubbing from both variable-centered and person-centered perspectives. The findings showed that phubbing increased on average across the three waves, but adolescents followed heterogeneous developmental trajectories. Mobile phone usage duration was associated with higher initial levels of phubbing and with membership in trajectory groups characterized by higher baseline phubbing. These findings suggest that adolescent phubbing is a dynamic and heterogeneous behavior, and that future prevention and intervention efforts should consider both general developmental trends and subgroup-specific patterns.

## Data Availability

The datasets presented in this article are not readily available because the data supporting the findings of this study are available upon request from the corresponding author. However, access to the data is restricted due to privacy considerations and cannot be made publicly available. Requests to access the datasets should be directed to lishoufengnju@163.com.
